# Neurodevelopment at Age 9 Years Among Children Born at 32 to 36 Weeks’ Gestation

**DOI:** 10.1001/jamanetworkopen.2024.45629

**Published:** 2024-11-18

**Authors:** Jeanie L. Y. Cheong, Rheanna M. Mainzer, Lex W. Doyle, Joy E. Olsen, Rachel Ellis, Tara L. FitzGerald, Kate L. Cameron, Lauren Rossetti, Peter J. Anderson, Alicia J. Spittle

**Affiliations:** 1 Clinical Sciences, Murdoch Children’s Research Institute, Melbourne, Victoria, Australia; 2 Neonatal Services, Royal Women’s Hospital, Melbourne, Victoria, Australia; 3 Department of Obstetrics, Gynaecology and Newborn Health, University of Melbourne, Melbourne, Victoria, Australia; 4 Department of Paediatrics, University of Melbourne, Melbourne, Victoria, Australia; 5 School of Psychological Sciences, Turner Institute for Brain & Mental Health, Monash University, Melbourne, Victoria, Australia; 6 Clinical Epidemiology and Biostatistics Unit, Murdoch Children’s Research Institute, Melbourne, Victoria, Australia; 7 Department of Physiotherapy, University of Melbourne, Melbourne, Victoria, Australia

## Abstract

**Importance:**

Although children born moderate to late preterm (MLP; 32-36 weeks’ gestation) have more neurodevelopmental problems compared with children born early term or later (≥37 weeks’ gestation), detailed understanding of affected domains at school age is lacking. Little is known of risk factors for poorer development.

**Objective:**

To examine whether being born MLP compared with being born early term or later is associated with neurodevelopmental outcomes at age 9 years and to describe factors associated with poorer neurodevelopment in children born MLP.

**Design, Setting, and Participants:**

This prospective, longitudinal cohort study recruited children born MLP and children born early term or later with healthy birth weight (≥2500 g) at a single tertiary hospital in Melbourne, Victoria, Australia, between December 7, 2009, and March 26, 2014. Nine-year follow-up occurred between June 20, 2019, and February 27, 2024.

**Exposure:**

Moderate to late preterm birth.

**Main Outcomes and Measures:**

Cognitive ability, academic performance, motor function, behavior, and social communication skills, assessed at 9-year follow-up. Group differences were estimated using linear, logistic, or quantile regression adjusted for multiple birth and socioeconomic risk. Multiple imputation was used to account for missing data. Associations of antenatal and neonatal factors and developmental delay at 2 years with poorer 9-year neurodevelopment were explored using univariable regression.

**Results:**

Of 201 recruited children born MLP and 201 born early term or later, 159 born MLP (79.1%; 72 [45.3%] male) and 137 born early term or later (68.2%; 75 [54.7%] male) were assessed. Compared with children born early term or later, children born MLP had lower mean (SD) full-scale IQ scores (105.2 [13.6] vs 110.1 [13.0]; adjusted mean difference, −4.4 [95% CI, −7.7 to −1.0]) and poorer performance for cognitive domains, including verbal comprehension, visuospatial, and working memory. They also had poorer academic performance: pseudoword decoding (mean [SD] score, 103.0 [11.3] vs 107.3 [10.5]; adjusted mean difference, −4.0 [95% CI, −7.0 to −1.1]) and mathematics (mean [SD] score, 96.6 [14.7] vs 101.5 [14.5]; adjusted mean difference, −5.0 [95% CI, −8.8 to −1.2]). Children born MLP had similar manual dexterity to those born early term or later (mean [SD] score, 8.4 [3.5] vs 9.1 [3.4]; adjusted mean difference, −0.9 [95% CI, −1.8 to 0.04]) but more behavioral difficulties (50 of 158 [31.7%] vs 29 of 135 [21.5%]; adjusted risk ratio, 1.57 [95% CI, 1.06-2.33]). Developmental delay at 2 years was associated with poorer 9-year neurodevelopment across multiple domains.

**Conclusions and Relevance:**

In this longitudinal cohort study of children born MLP, neurodevelopmental challenges persisted into school age. An assessment at age 2 years may assist in identifying children born MLP who are at risk of school-age impairments.

## Key Points

**Question:**

Is moderate to late preterm (MLP) birth (32 to 36 weeks’ gestation) associated with neurodevelopment at school age?

**Findings:**

In this cohort study of 159 children born MLP and 137 born early term or later (≥37 weeks’ gestation) recruited at birth, children born MLP had poorer scores in cognitive and academic performance and more behavior difficulties at age 9 years than children born early term or later.

**Meaning:**

The findings suggest that developmental surveillance to school age is important for children born MLP.

## Teaser

This cohort study investigates whether moderate to late preterm birth (32 to 36 weeks’ gestation) is associated with neurodevelopmental outcomes at age 9 years among children in Australia.

## Introduction

Moderate to late preterm (MLP) birth (32-36 weeks’ gestation) accounts for 85% of preterm birth (<37 weeks’ gestation), totaling 11.4 million births worldwide in 2020.^1^ Recent research has refuted past misconceptions that MLP birth was associated with little to no increase in developmental delays compared with full-term birth.^2,3^ Given the large number of children born MLP, even small increases in developmental delays could have substantial effects on health and educational resources.

There are limitations of current research. Most studies report developmental outcomes in the first few years after birth,^4,5,6^ which are only moderately predictive of outcomes at school age,^7^ a critical time in a child’s development during which specific cognitive and academic skills begin to emerge. The few studies reporting outcomes at school age have focused on broad summary scales^3,8^ without detailed characterization of the underlying neurodevelopmental skills. Knowledge of the specific skills affected is critical for designing interventions to improve outcomes for children born MLP. Furthermore, information on early-life risk factors that predict poorer outcomes at school age for children born MLP is lacking.^9^ Risk factors identified for children born at less than 32 weeks’ gestation, such as neonatal brain injury, bronchopulmonary dysplasia, and neonatal surgery, rarely occur in children born MLP.^10^ Identifying early-life risk factors for poorer development at school age will help prioritize surveillance programs and access to early intervention.

We sought to address these knowledge gaps by estimating whether being born MLP compared with being born early term or later (≥37 weeks’ gestation) is associated with cognitive ability, academic performance, motor function, behavior, and social communication skills at 9 years of age. We also aimed to identify factors in the newborn period and infancy that were associated with poorer neurodevelopment at 9 years of age. We hypothesized that compared with children born early term or later, those born MLP would have worse outcomes in all reported domains of development at 9 years of age. We also hypothesized that earlier gestational age, male sex, higher socioeconomic risk, and developmental delay at 2 years of age would be associated with poorer development at 9 years of age.

## Methods

### Participants

LaPrem is a longitudinal cohort study of children born MLP and children born early term or later with healthy birth weight (≥2500 g) who were recruited after birth from the Royal Women’s Hospital, a tertiary hospital in Melbourne, Victoria, Australia, between December 7, 2009, and March 26, 2014. Children with congenital abnormalities or genetic syndromes known to affect development were excluded. In addition, infants born early term or later were excluded if they were unwell at birth, received resuscitation, or were admitted to the neonatal nursery. Children were previously assessed at age 2 years.^4,11^ At 9 years’ corrected age, children underwent a 2-day assessment, between June 20, 2019, and February 27, 2024.^12^ Follow-up of participants was limited during 2020 and 2021 due to extensive COVID-19 pandemic lockdowns in Victoria, Australia. Ethical approval was provided by the human research ethics committees of the Royal Women’s Hospital and the Royal Children’s Hospital Melbourne. All parents gave written informed consent. The study protocol has been published elsewhere.^12^ This report followed the Strengthening the Reporting of Observational Studies in Epidemiology (STROBE) reporting guideline for cohort studies.

### Perinatal Data

Perinatal, neonatal, and maternal data were collected prospectively. Socioeconomic risk was assessed at the 9-year follow-up using 6 variables: family structure, educational level of the primary caregiver, employment status and occupation of the primary income earner, language spoken at home, and maternal age at birth of the child.^13^ Each variable was scored on a 3-point scale (from 0 for lowest risk to 2 for highest risk), summed to give a total score, and dichotomized to higher (total score ≥2) or lower (total score <2) socioeconomic risk.

### Outcome Measures

Children were assessed by trained assessors (R.E., T.L.F., K.L.C., L.R) unaware of birth status or clinical history. Age was corrected for prematurity to account for a known bias in cognitive test scores.^14^ In addition to the formal assessments, parents were asked whether their child had a developmental disability, including cerebral palsy, autism spectrum disorder (ASD), and attention-deficit/hyperactivity disorder (ADHD).

Cognitive ability was measured using the Wechsler Intelligence Scale for Children (WISC), Fifth Edition: Australian and New Zealand Standardized Edition.^15^ Full-scale IQ (FSIQ) was used as a measure of general intelligence, with age-standardized index scores from 5 domains of cognitive functioning: verbal comprehension, visuospatial, fluid reasoning, working memory, and processing speed. Intellectual impairment was defined as a score less than −1 SD (15 points) relative to the mean for the children born early term or later.

Reading (single-word reading and pseudoword decoding), spelling, and mathematics skills were assessed using the Wechsler Individual Achievement Test, Third Edition: Australian and New Zealand Standardized Edition.^16^ Impairments in reading, spelling, and mathematics were defined as less than −1 SD (15 points) relative to the mean for the group born early term or later.

Motor function was assessed using the Movement Assessment Battery for Children–Second Edition (MABC-2).^17^ The overall MABC-2 standard score and 3 subscales (balance, aiming and catching, and manual dexterity) have a mean (SD) score of 10 (3), with higher scores indicating better performance. Motor impairment was defined as either cerebral palsy or MABC-2 score less than or equal to the fifth centile.

Behavior was assessed using the Strengths and Difficulties Questionnaire (SDQ),^18^ a parent- or primary caregiver–reported measure that assesses 5 scales relating to emotional symptoms, conduct problems, hyperactivity and inattention, peer relationship problems, and prosocial behavior. Higher scores from the first 4 scales indicate more behavioral problems and were summed to a total difficulties score (range, 0-40). Any behavioral difficulty was defined as having a total difficulties score in the 80th percentile or higher based on Australian norms.^19^

Social communication was assessed using the Lifetime form of the Social Communication Questionnaire (SCQ),^20^ a parent- or primary caregiver–reported measure evaluating reciprocal social interaction, communication, and repetitive and/or stereotypic behavior. Higher scores indicate more behaviors associated with autism. Scores greater than the cutoff of 15 points indicate a higher likelihood of ASD,^21^ with recommendations for further diagnostic assessment.

Some children did not have impairment in any of the reported domains. For these children, we computed a composite outcome of no impairment.

### Statistical Analysis

Data were analyzed using Stata, version 18 (StataCorp LLC). Participant characteristics were summarized as mean (SD) or number (percentage) and compared between those who were assessed at 9 years and those who were not. Continuous outcomes at 9 years were compared between groups using mean or median differences estimated using linear or quantile regression, respectively. Binary outcomes were compared between groups using risk ratios (RRs) and risk differences (RDs) estimated using generalized linear models with a binomial family and either a log (RRs) or an identity (RDs) link function. A separate model was used for each developmental outcome. All models were adjusted for multiple birth and socioeconomic risk at 9 years, and except for quantile regressions, they were fitted using generalized estimating equations with an exchangeable correlation structure to account for clustering of multiple births within the same family and robust SEs. The directed acyclic graph in eFigure 1 in Supplement 1 depicts the assumptions made for this analysis.

To account for missing data, estimates were obtained using multiple imputation by chained equations; assumptions and a justification for the use of multiple imputation are provided in eFigure 2 in Supplement 1. A separate multiple imputation procedure was conducted for each outcome. All imputation models included gestational age group, multiple birth, socioeconomic risk, perinatal and neonatal variables, disability status at 2 years (cerebral palsy and/or developmental delay, defined as a score <−1 SD below the mean for children born early term or later on either the cognitive, language, or motor domains on the Bayley Scales of Infant and Toddler Development, Third Edition [Bayley-III]),^22^ and the relevant outcome. Forty imputed datasets were created, and the resulting inference was combined using Rubin rules.^23^ We also reported results from a complete case analysis.

Within the group born MLP, associations between early-life variables and 9-year outcomes were explored using univariable logistic or linear regression fitted using generalized estimating equations with an exchangeable correlation structure to account for clustering of multiple births within the same family and robust SEs. The variables were selected a priori and included assisted conception, antenatal corticosteroids, gestational age, multiple birth, male sex, birth weight *z* score, respiratory support, higher socioeconomic risk at birth, and developmental delay at 2 years of age (defined as a score <−1 SD below the mean for children born early term or later on either the cognitive, language, or motor domains on the Bayley-III). Given the multiple comparisons, we interpreted our findings by focusing on overall patterns and magnitude of differences rather than on individual *P* values.

## Results

### Participant Characteristics

**Figure 1.**
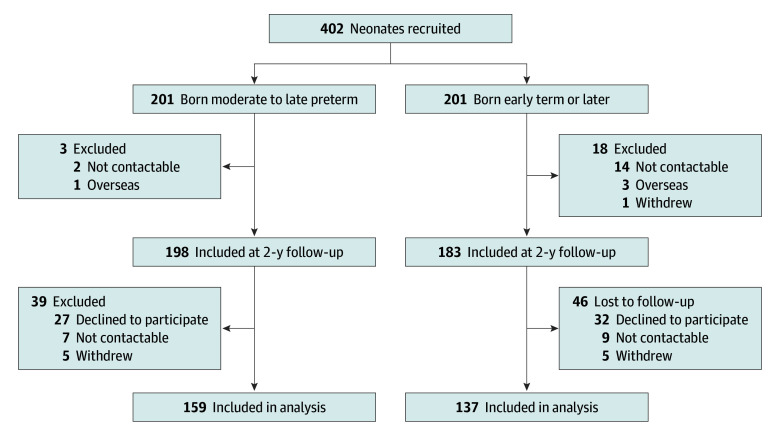
Participant Recruitment and Follow-Up

Of the 201 children born MLP and 201 born early term or later who were recruited at birth, data at 9 years were available for 159 born MLP (79.1%; 87 [54.7%] female and 72 [45.3%] male) and 137 born early term or later (68.2%; 62 [45.3%] female and 75 [54.7%] male) (Figure 1). There were group differences in perinatal characteristics related to prematurity (Table 1). Compared with children born early term or later, children born MLP were more likely to be born following assisted conception, in a multiple birth, and/or via cesarean delivery; have lower birth weight *z* scores and higher socioeconomic risk; and have received respiratory support after birth. There were no children in either group diagnosed with cerebral palsy, blindness, or deafness at 9 years. In both groups, mothers of participants who were assessed at 9 years were older, and the proportion of participants with higher socioeconomic risk was lower compared with those who were not assessed at 9 years (eTable 1 in Supplement 1). In the group born MLP, the proportions of participants exposed to antenatal corticosteroids, male participants, and those from a multiple birth were lower among those who were assessed at 9 years than among those who were not. In the group born early term or later, the rate of assisted conception was almost double among those who were assessed at 9 years compared with those who were not.

**Table 1.** Participant Characteristics

Variable	Participants^a^	
	Born MLP (n = 159)	Born ≥early term (n = 137)
**Perinatal and neonatal period**		
Maternal age, mean (SD), y	34.3 (4.7)	33.7 (4.6)
Maternal preeclampsia	29 (18.2)	2 (1.5)
Assisted conception, No./total No.	31/156 (19.9)	16/137 (11.7)
Gestational age at birth, mean (SD), wk	34.3 (1.2)	39.9 (1.2)
Antenatal corticosteroid exposure	94 (59.1)	4 (2.9)
Antenatal magnesium sulfate, No./total No.	12/159 (7.6)	0/136 (0)
Multiple birth	55 (34.6)	2 (1.5)
Cesarean delivery	110 (69.2)	51 (37.2)
Sex		
Female	87 (54.7)	62 (45.3)
Male	72 (45.3)	75 (54.7)
Birth weight, mean (SD), g	2156 (436)	3567 (455)
Birth weight *z* score, mean (SD)	−0.29 (0.97)	0.25 (0.84)
Apgar score at 5 min, median (IQR)	9 (8-9)	9 (9-9)
Any respiratory support	22 (13.8)	0
Brain injury, No./total No.^b^	0/159	0/69
Neonatal hospitalization		
No. (%)	151 (95.0)	135 (98.5)
Median (IQR), d	21 (14-28)	3 (2-3)
Neurodevelopmental delay at 2 y^c^	73 (45.9)	37/131 (28.2)
**9-y Follow-up**		
Higher socioeconomic risk at 9 y	39 (24.5)	23 (16.8)
Corrected age at follow-up, mean (SD), y	9.7 (0.4)	9.8 (0.4)
Cerebral palsy	0	0
Autism spectrum disorder diagnosed, No./total No.	13/144 (9.0)	8/129 (6.2)
Attention-deficit/hyperactivity disorder diagnosed, No./total No.	16/144 (11.1)	7/129 (5.4)
Repeated a school year, No./total No.	5/157 (3.2)	0/132 (0)

Abbreviation: MLP, moderate to late preterm.

^a^ Data are presented as number or number/total number (percentage) unless otherwise indicated. MLP birth was 32 to 36 weeks’ gestation and early term or later, 37 or more weeks’ gestation.

^b^ Any intraventricular hemorrhage or cystic periventricular leukomalacia diagnosed based on cranial ultrasonography or brain magnetic resonance imaging (the latter was part of the research protocol for neonatal imaging).

^c^ Defined as any cerebral palsy or any developmental delay defined as a score less than −1 SD below the mean for children born early term or later on either the cognitive, language, or motor domains on the Bayley Scales for Infant and Toddler Development, Third Edition.

### Comparison of Outcomes Between Groups at 9 Years

Children born MLP had lower mean (SD) FSIQ scores than children born early term or later (105.2 [13.6] vs 110.1 [13.0]), with an adjusted mean difference of −4.4 points (95% CI, −7.7 to −1.0 points), which is equivalent to −0.3 SD (Table 2 and eFigure 3 in Supplement 1). Similar results were found for most other cognitive domains, with the greatest differences in verbal comprehension, visuospatial, and working memory indices. Children born MLP performed less well in reading (mean [SD] pseudoword decoding score, 103.0 [11.3] vs 107.3 [10.5]) and mathematics (mean [SD] score, 96.6 [14.7] vs 101.5 [14.5]) compared with children born early term or later, with adjusted mean differences of −4.0 points (95% CI, −7.0 to −1.1 points) and −5.0 points (95% CI, −8.8 to −1.2 points), respectively, which are in the range of −0.3 SD. The overall standard scores for the MABC-2 were similar in the group born MLP and in children born early term or later although among those born MLP, and performance in the manual dexterity scale was similar (mean [SD] score, 8.4 [3.5] vs 9.1 [3.4]; adjusted mean difference, −0.9 [95% CI, −1.8 to 0.04]). Hyperactivity and inattention scores were slightly higher among the children born MLP compared with children born early term or later.

**Table 2.** Comparison of Neurodevelopmental Outcome Scores at 9 Years Between Children Born MLP and Children Born Early Term or Later^a^

Measure	MLP birth		Early term birth or later		Adjusted mean or median difference (95% CI)^c^
	Score^b^	Participants, No.	Score^b^	Participants, No.	
General intelligence					
Full-scale IQ	105.2 (13.6)	155	110.1 (13.0)	129	−4.4 (−7.7 to −1.0)
Verbal comprehension index	109.6 (12.7)	156	113.7 (13.4)	129	−3.6 (−7.2 to −0.1)
Visuospatial index	100.8 (13.9)	119	107.6 (14.6)	117	−5.3 (−9.6 to −1.0)
Fluid reasoning index	104.8 (13.8)	157	108.0 (13.8)	129	−3.0 (−6.3 to 0.3)
Working memory index	103.1 (12.8)	152	107.4 (12.3)	129	−4.6 (−8.0 to −1.1)
Processing speed index	98.4 (14.0)	113	100.0 (12.4)	104	−1.5 (−5.5 to 2.6)
Academic performance					
Reading					
Single word	106.1 (15.2)	155	110.0 (13.4)	129	−3.1 (−6.7 to 0.5)
Pseudoword decoding	103.0 (11.3)	155	107.3 (10.5)	129	−4.0 (−7.0 to −1.1)
Spelling	103.1 (16.2)	154	106.1 (14.8)	125	−2.4 (−6.2 to 1.3)
Mathematics	96.6 (14.7)	148	101.5 (14.5)	120	−5.0 (−8.8 to −1.2)
Motor function					
MABC-2 standard score	8.7 (3.1)	142	9.1 (3.3)	118	−0.7 (−1.5 to 0.1)
Balance	8.4 (3.1)	142	9.1 (3.4)	119	−0.1 (−1.1 to 0.9)
Aiming and catching	10.2 (2.8)	148	10.2 (2.7)	123	−0.2 (−0.9 to 0.5)
Manual dexterity	8.4 (3.5)	142	9.1 (3.4)	119	−0.9 (−1.8 to 0.04)
Behavioral problems					
Emotional symptoms, median (IQR)	2 (1-5)	158	2 (1-4)	135	0 (−0.7 to 0.7)
Conduct problems, median (IQR)	1 (0-2)	158	1 (0-2)	135	0 (−0.4 to 0.4)
Hyperactivity and inattention	3.9 (3.0)	158	3.4 (2.8)	135	0.9 (0.2 to 1.5)
Peer relationship problems, median (IQR)	1 (0-2)	158	1 (0-2)	135	0 (−0.5 to 0.5)
Prosocial behavior, median (IQR)	9 (8-10)	158	9 (7-10)	135	0.2 (−0.8 to 1.1)
Total difficulties score	9.5 (6.4)	158	8.7 (5.8)	135	1.4 (−0.2 to 2.9)
Social communication: total SCQ score, median (IQR)	2.5 (1-6)	156	3 (1-6)	131	0.2 (−1.0 to 1.3)

Abbreviations: MLP, moderate to late preterm; MABC-2, Movement Assessment Battery for Children, Second Edition; SCQ, Social Communication Questionnaire.

^a^ MLP birth was 32 to 36 weeks’ gestation and early term or later, 37 or more weeks’ gestation.

^b^ Scores are presented as mean (SD) unless otherwise indicated.

^c^ Estimated using multiple imputation; regression models were adjusted for socioeconomic risk and multiple pregnancy as informed by the directed acyclic graph (eFigure 1 in Supplement 1).

For dichotomous outcomes, the number of children with no impairment was similar between groups, with an adjusted RD of −8.9% (95% CI, −21.7% to 3.9%). The rate of any academic impairment in the group born MLP was similar to that among children born early term or later (adjusted RD, 6.4%; 95% CI, −6.8% to 19.7%) (Table 3). Rates of impairment in cognition, motor, or communication problems were similar between children born MLP and those born early term or later. However, the group born MLP had more behavioral difficulties than children born early term or later (50 of 158 [31.7%] vs 29 of 135 [21.5%]), with an overall adjusted RR of 1.57 (95% CI, 1.06-2.33) and adjusted RD of 12.8% (95% CI, 2.1%-23.5%). Of the 16 children identified as at risk of autism on the SCQ, all but 2 (12.5%; 1 in each group) had received a diagnosis of ASD. The complete case analyses had similar conclusions (eTables 2 and 3 in Supplement 1).

**Table 3.** Impairment at 9 Years in Children Born MLP Compared With Children Born at Early Term or Later

Outcome	Participants, No./total No. (%)^a^		Adjusted RR (95% CI)^b^	Adjusted RD, % (95% CI)^b^
	Born MLP	Born ≥early term		
No impairment^c^	60/159 (37.7)	63/136 (46.3)	0.81 (0.59-1.10)	−8.9 (−21.7 to 3.9)
General intelligence				
Full-scale IQ <−1 SD^d^	30/155 (19.4)	21/129 (16.3)	1.20 (0.72-2.02)	3.2 (−6.3 to 12.7)
Academic performance				
Reading <−1 SD^d^				
Single word	34/155 (21.9)	21/129 (16.3)	1.31 (0.79-2.16)	5.2 (−4.7 to 15.2)
Pseudoword decoding	27/155 (17.4)	12/129 (9.3)	1.58 (0.85-2.95)	6.0 (−2.3 to 14.3)
Spelling <−1 SD^d^	38/154 (24.7)	22/125 (17.6)	1.30 (0.81-2.09)	5.2 (−5.0 to 15.4)
Mathematics <−1 SD^d^	36/148 (24.3)	18/120 (15.0)	1.57 (0.91-2.71)	8.2 (−2.0 to 18.4)
Any academic impairment^e^	68/151 (45.0)	48/121 (39.7)	1.16 (0.85-1.59)	6.4 (−6.8 to 19.7)
Motor impairment^f^	20/142 (14.1)	18/118 (15.3)	1.10 (0.62-1.97)	1.6 (−7.6 to 10.9)
Any behavioral difficulties	50/158 (31.7)	29/135 (21.5)	1.57 (1.06-2.33)	12.8 (2.1 to 23.5)
Social communication: at risk of autism^g^	9/156 (5.8)	7/131 (5.3)	1.44 (0.56-3.73)	2.6 (−4.0 to 9.3)

Abbreviations: DCD, developmental coordination disorder; MLP, moderate to late preterm; RD, risk difference; RR, risk ratio.

^a^ MLP birth was 32 to 36 weeks’ gestation and early term or later, 37 or more weeks’ gestation.

^b^ Estimated using multiple imputation; regression models were adjusted for socioeconomic risk and multiple pregnancy as informed by the directed acyclic graph (eFigure 1 in Supplement 1).

^c^ No impairment in cognitive, academic performance, motor, behavior, or social communication measures, as defined in the text.

^d^ Compared with children born early term or later.

^e^ Impairment in any of the 4 academic domains (single-word reading, pseudoword decoding, spelling, or mathematics).

^f^ Any cerebral palsy or Movement Assessment Battery for Children–Second Edition score less than or equal to the fifth centile (there were no children with cerebral palsy in either group).

^g^ Social Communication Questionnaire score of 15 or higher.

### Variables Associated With Outcomes at 9 Years

**Figure 2.**
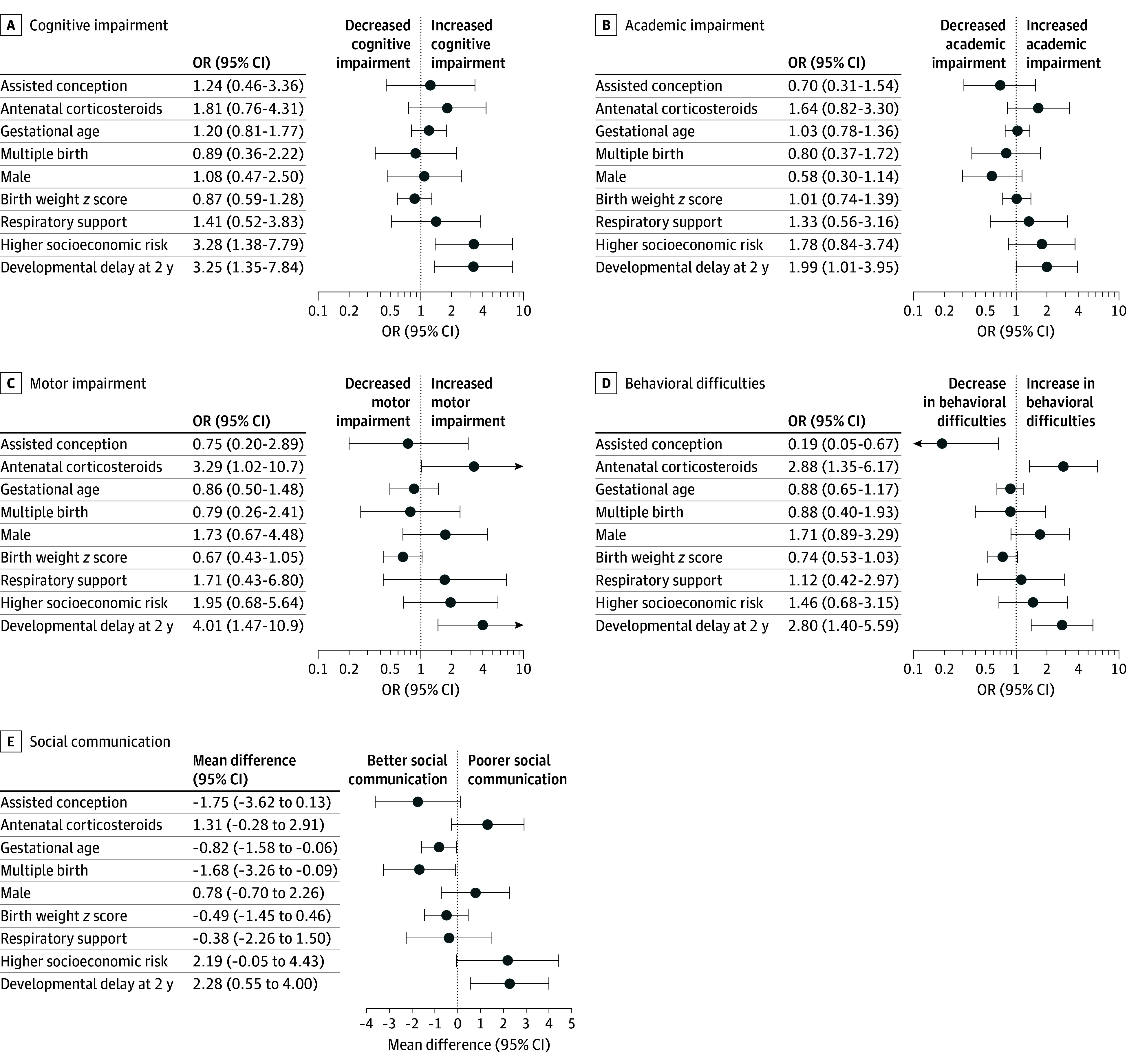
Univariable Associations Between Early-Life Factors and 9-Year Outcomes Among Children Born at 32 to 36 Weeks’ Gestation

Developmental delay at 2 years of age was associated with impairments in all developmental domains at age 9 years (Figure 2 and eTable 4 in Supplement 1). Receiving antenatal corticosteroids was associated with motor impairment and behavioral difficulties. Higher socioeconomic risk was associated with cognitive impairment and poorer social communication. However, a higher gestational age and multiple birth were associated with better social communication.

## Discussion

The major findings of this cohort study are that children born MLP had lower scores in multiple neurodevelopmental domains and more behavioral difficulties at 9 years of age compared with their peers born early term or later. Differences were greatest for general cognitive ability, reading (pseudoword decoding) and mathematics, manual dexterity, and hyperactivity and inattention. The findings at 9 years are consistent with cognitive, language, and motor delays reported at 2 years’ corrected age in the group born MLP compared with children born early term or later.^4^ We chose a cutoff of less than −1 SD because even mild developmental delay affects function and children with mild delay may benefit from intervention. The current findings add to the growing evidence that the developmental challenges associated with MLP birth persist to school age. We also identified that developmental delay at 2 years was associated with a range of adverse outcomes at 9 years in children born MLP. This suggests that developmental assessment at 2 years of age is important to identify the children born MLP who may have high risk of neurodevelopmental and behavioral problems at school age.

Nonetheless, most children born MLP scored within −2 SD of the reference mean score of 100 for FSIQ and all academic domains (eFigure 3 in Supplement 1). We reported mean differences in the range of −0.3 SD for general cognition, which is considered to be a clinically important difference and is of similar magnitude to the standardized mean differences pooled from 4 studies of children born MLP with ages ranging from 6 to 13 years.^9,24,25,26,27^ The group born early term or later in our study had a mean FSIQ of 110.1, higher than published norms, which may reflect selection of a higher-functioning group or a Flynn effect (the tendency for IQ scores to drift upward over time) given that the version of the WISC used in our study was published in 2016. As differences of similar magnitude were found in most cognitive domains, including verbal comprehension, visuospatial, fluid reasoning, and working memory, the association of MLP birth with cognition is unlikely to be specific to selective cognitive skills.

The group born MLP had lower academic performance scores than children born early term or later in pseudoword decoding and mathematics in the order of a clinically important difference of −0.3 SD. The rates of any impairment in academic performance were similar in the group born MLP than children born early term or later. Lower educational achievement at 6 to 11 years of age has been reported in children born MLP compared with those born early term or later, with pooled relative risk estimates of 1.96 (95% CI, 1.11-3.43) for those born at 32 to 33 weeks’ gestation and 1.21 (95% CI, 1.10-1.32) for those born at 34 to 36 weeks’ gestation.^24,28,29,30,31^ Difficulties in mathematics have been reported in children born very preterm compared with children born early term or later,^32,33^ although this has been less studied in groups born MLP. A linkage study in California reported poorer mathematics proficiency from grades 3 to 8, which included children born MLP of similar ages to those in our study.^34^ Knowing which domains of academic performance are most affected may help tailor interventions for children born MLP.

There were lower MABC-2 scores in the manual dexterity subscale among children born MLP. However, rates of motor impairment were similar between groups. Of interest, the group born early term or later had mean MABC-2 standard scores that were lower than test norms, and a relatively high rate scored at or below the fifth centile. The reasons for this are unclear and warrant further investigation. For other developmental domains, the children born early term or later had higher means than the test norms, similar to previous findings in which Australian groups born early term or later had higher means than published test norms.^32,35^

We reported higher rates of behavioral difficulties using the SDQ in the group born MLP compared with children born early term or later. Pettinger et al^24^ reported ADHD diagnosis or ADHD symptom prevalence of 34.9 (95% CI, 33.6-36.3) per 1000 children in the group born MLP compared with 26.6 (95% CI, 26.3-26.8) per 1000 children among those born early term or later. There was a slight increase in relative risk for ASD in children born MLP compared with children born early term or later.^24^ Our study did not formally assess for ASD but instead classified children at risk of ASD based on an assessment of social communication. All but 2 children who were at risk of ASD had a previous diagnosis of ASD. The sample size in our study was not powered to detect group differences in either ADHD or ASD, although the sample had relatively high rates for both diagnoses.

We identified few early-life factors that were associated with 9-year neurodevelopment, apart from developmental delay at 2 years of age. Antenatal corticosteroid administration was associated with motor impairment and behavior difficulties. This concurs with recent concerns about the potential negative developmental association between antenatal corticosteroids and late preterm birth.^36^ The lack of associations between gestational age and many outcomes at 9 years suggests that there is little difference in risk for children born within the 32- to 36-week gestational age range. Our study supports the importance of a developmental assessment at 2 years of age to identify children born MLP who may have high risk of later neurodevelopmental problems. As the absolute numbers of children born MLP were large, screening tools may be appropriate as a first-line approach to stratify risk.

### Strengths and Limitations

Our study’s strengths include recruitment of participants at birth, with detailed perinatal, neonatal, and early infancy data. We conducted a broad suite of direct assessments by assessors blinded to participant group and clinical history, rather than relying on diagnostic categories; this enabled deep phenotyping of deficits that contributed to neurodevelopmental problems reported by other studies in a meta-analysis.^24^ This information may be valuable to clinicians caring for children born MLP and also for development of targeted interventions.

This study also has limitations. Our retention rates at 9 years were lower than at the 2-year follow-up, in part due to disruptions with COVID-19 lockdowns in Victoria, Australia. We used multiple imputation informed by directed acyclic graphs for estimation in the presence of missing data. The single tertiary center cohort may represent children who were sicker at birth and thus at higher risk of developmental delays compared with the whole population of children born MLP.

## Conclusions

The findings of this cohort study suggest that neurodevelopmental problems in a broad range of domains in children born MLP may persist at school age. Neurodevelopmental status at 2 years was associated with longer-term outcomes. Thus, consideration should be given to developmental screening at 2 years of age for children born MLP. Further research for an in-depth understanding of affected developmental domains is important to develop targeted interventions for this large and growing group of children.

## Notes

Supplement 1. **eTable 1.** Comparison Between Participants Who Were Seen at 9 Years and Those Who Were Not **eTable 2.** Complete Case Analyses of Neurodevelopmental Outcome Scores at 9 Years Among Children Born MLP Compared With Term-Born Children **eTable 3.** Complete Case Analyses of Impairment Rates at 9 Years Among Children Born MLP Compared With Term-Born Children **eTable 4.** Univariable Associations Between Early-Life Factors and Outcomes at 9 Years for Children Born MLP **eFigure 1.** Directed Acyclic Graph for 9-Year Outcomes Depicting Assumed Causal Relationships Between Variables **eFigure 2.** Missingness-Directed Acyclic Graph Depicting the Assumptions Made About the Causes of Missing Data **eFigure 3.** Cognitive and Academic Scores by Birth Group

Supplement 2.
Data Sharing Statement

## References

[1] Ohuma EO, Moller AB, Bradley E, . National, regional, and global estimates of preterm birth in 2020, with trends from 2010: a systematic analysis. Lancet. 2023;402(10409):1261-1271. doi:10.1016/S0140-6736(23)00878-4 37805217

[2] Woythaler M. Neurodevelopmental outcomes of the late preterm infant. Semin Fetal Neonatal Med. 2019;24(1):54-59. doi:10.1016/j.siny.2018.10.002 30322826

[3] Mitha A, Chen R, Razaz N, . Neurological development in children born moderately or late preterm: national cohort study. BMJ. 2024;384:e075630. doi:10.1136/bmj-2023-075630 38267070 PMC11957549

[4] Cheong JL, Doyle LW, Burnett AC, . Association between moderate and late preterm birth and neurodevelopment and social-emotional development at age 2 years. JAMA Pediatr. 2017;171(4):e164805. doi:10.1001/jamapediatrics.2016.4805 28152144

[5] Woythaler MA, McCormick MC, Smith VC. Late preterm infants have worse 24-month neurodevelopmental outcomes than term infants. Pediatrics. 2011;127(3):e622-e629. doi:10.1542/peds.2009-3598 21321024

[6] Johnson S, Evans TA, Draper ES, . Neurodevelopmental outcomes following late and moderate prematurity: a population-based cohort study. Arch Dis Child Fetal Neonatal Ed. 2015;100(4):F301-F308. doi:10.1136/archdischild-2014-307684 25834170 PMC4484499

[7] Roberts G, Anderson PJ, Doyle LW; Victorian Infant Collaborative Study Group. The stability of the diagnosis of developmental disability between ages 2 and 8 in a geographic cohort of very preterm children born in 1997. Arch Dis Child. 2010;95(10):786-790. doi:10.1136/adc.2009.160283 19828882

[8] Chan E, Quigley MA. School performance at age 7 years in late preterm and early term birth: a cohort study. Arch Dis Child Fetal Neonatal Ed. 2014;99(6):F451-F457. doi:10.1136/archdischild-2014-306124 24966128

[9] Cserjesi R, Van Braeckel KNJA, Butcher PR, . Functioning of 7-year-old children born at 32 to 35 weeks’ gestational age. Pediatrics. 2012;130(4):e838-e846. doi:10.1542/peds.2011-2079 22945414

[10] Cheong JLY, Lee KJ, Boland RA, ; Victorian Infant Collaborative Study Group. Changes in long-term prognosis with increasing postnatal survival and the occurrence of postnatal morbidities in extremely preterm infants offered intensive care: a prospective observational study. Lancet Child Adolesc Health. 2018;2(12):872-879. doi:10.1016/S2352-4642(18)30287-6 30361130

[11] Spittle AJ, Walsh JM, Potter C, . Neurobehaviour at term-equivalent age and neurodevelopmental outcomes at 2 years in infants born moderate-to-late preterm. Dev Med Child Neurol. 2017;59(2):207-215. doi:10.1111/dmcn.13297 27775148

[12] Cheong J, Cameron KLI, Thompson D, . Impact of moderate and late preterm birth on neurodevelopment, brain development and respiratory health at school age: protocol for a longitudinal cohort study (LaPrem study). BMJ Open. 2021;11(1):e044491. doi:10.1136/bmjopen-2020-044491 33518527 PMC7852967

[13] Roberts G, Howard K, Spittle AJ, Brown NC, Anderson PJ, Doyle LW. Rates of early intervention services in very preterm children with developmental disabilities at age 2 years. J Paediatr Child Health. 2008;44(5):276-280. doi:10.1111/j.1440-1754.2007.01251.x 17999667

[14] Wilson-Ching M, Pascoe L, Doyle LW, Anderson PJ. Effects of correcting for prematurity on cognitive test scores in childhood. J Paediatr Child Health. 2014;50(3):182-188. doi:10.1111/jpc.12475 24617343

[15] Wechsler D. *Wechsler Intelligence Scale of Children, Fifth Edition: Australian and New Zealand Standardised Edition (WISC-V A&NZ)*. Pearson; 2016.

[16] Joshua N, Wilson C, Bendrups N. *Wechsler Individual Achievement Test, Third Edition: Australian and New Zealand Standardised (WIAT-III A&NZ)*. Pearson; 2016.

[17] Henderson SE, Sugden DA. Movement Assessment Battery for Children. 2nd ed. The Psychological Corporation/Harcourt Brace Jovanovich; 2007, MABC-2.

[18] Goodman A, Goodman R. Strengths and Difficulties Questionnaire as a dimensional measure of child mental health. J Am Acad Child Adolesc Psychiatry. 2009;48(4):400-403. doi:10.1097/CHI.0b013e3181985068 19242383

[19] Youth in Mind. Welcome to the SDQ. Accessed August X, 2024. https://sdqscore.org/

[20] Rutter M, Bailey A, Lord C. The Social Communication Questionnaire. WPS; 2003.

[21] Berument SK, Rutter M, Lord C, Pickles A, Bailey A. Autism screening questionnaire: diagnostic validity. Br J Psychiatry. 1999;175:444-451. doi:10.1192/bjp.175.5.444 10789276

[22] Bayley N. The Bayley Scales of Infant Development. 3rd ed. Psychological Corporation; 2005.

[23] Rubin DB. Multiple Imputation for Nonresponse in Surveys. Wiley; 1987. doi:10.1002/9780470316696

[24] Pettinger KJ, Copper C, Boyle E, Blower S, Hewitt C, Fraser L. Risk of developmental disorders in children born at 32 to 38 weeks’ gestation: a meta-analysis. Pediatrics. 2023;152(6):e2023061878. doi:10.1542/peds.2023-061878 37946609 PMC10657778

[25] Talge NM, Holzman C, Wang J, Lucia V, Gardiner J, Breslau N. Late-preterm birth and its association with cognitive and socioemotional outcomes at 6 years of age. Pediatrics. 2010;126(6):1124-1131. doi:10.1542/peds.2010-1536 21098151

[26] Schneider LA, Burns NR, Giles LC, . Cognitive abilities in preterm and term-born adolescents. J Pediatr. 2014;165(1):170-177. doi:10.1016/j.jpeds.2014.03.030 24793204

[27] Pérez-Pereira M, Fernández MP, Gómez-Taibo ML, Martínez-López Z, Arce C. A follow-up study of cognitive development in low risk preterm children. Int J Environ Res Public Health. 2020;17(7):2380. doi:10.3390/ijerph17072380 32244477 PMC7178262

[28] Crockett LK, Ruth CA, Heaman MI, Brownell MD. Education outcomes of children born late preterm: a retrospective whole-population cohort study. Matern Child Health J. 2022;26(5):1126-1141. doi:10.1007/s10995-022-03403-8 35301671

[29] Libuy N, Gilbert R, Mc Grath-Lone L, Blackburn R, Etoori D, Harron K. Gestational age at birth, chronic conditions and school outcomes: a population-based data linkage study of children born in England. Int J Epidemiol. 2023;52(1):132-143. doi:10.1093/ije/dyac105 35587337 PMC9908051

[30] Richards JL, Chapple-McGruder T, Williams BL, Kramer MR. Does neighborhood deprivation modify the effect of preterm birth on children’s first grade academic performance? Soc Sci Med. 2015;132:122-131. doi:10.1016/j.socscimed.2015.03.032 25797101 PMC4400252

[31] Searle AK, Smithers LG, Chittleborough CR, Gregory TA, Lynch JW. Gestational age and school achievement: a population study. Arch Dis Child Fetal Neonatal Ed. 2017;102(5):F409-F416. doi:10.1136/archdischild-2016-310950 28154109

[32] Cheong JLY, Anderson PJ, Burnett AC, ; Victorian Infant Collaborative Study Group. Changing neurodevelopment at 8 years in children born extremely preterm since the 1990s. Pediatrics. 2017;139(6):e20164086. doi:10.1542/peds.2016-4086 28814550

[33] Clayton S, Simms V, Cragg L, . Etiology of persistent mathematics difficulties from childhood to adolescence following very preterm birth. Child Neuropsychol. 2022;28(1):82-98. doi:10.1080/09297049.2021.1955847 34472423

[34] Townley Flores C, Gerstein A, Phibbs CS, Sanders LM. Short-term and long-term educational outcomes of infants born moderately and late preterm. J Pediatr. 2021;232:31-37.e2. doi:10.1016/j.jpeds.2020.12.070 33412166

[35] Spittle AJ, Cameron K, Doyle LW, Cheong JL; Victorian Infant Collaborative Study Group. Motor impairment trends in extremely preterm children: 1991-2005. Pediatrics. 2018;141(4):e20173410. doi:10.1542/peds.2017-3410 29567814

[36] Ninan K, Liyanage SK, Murphy KE, Asztalos EV, McDonald SD. Evaluation of long-term outcomes associated with preterm exposure to antenatal corticosteroids: a systematic review and meta-analysis. JAMA Pediatr. 2022;176(6):e220483-e220483. doi:10.1001/jamapediatrics.2022.0483 35404395 PMC9002717

