# Supplementary material for: Neurodevelopment at Age 9 Years Among Children Born at 32 to 36 Weeks’ Gestation
Source: JAMA Netw Open. 2024 Nov 18;7(11):e2445629. doi: 10.1001/jamanetworkopen.2024.45629 (PMC11574691; doi:10.1001/jamanetworkopen.2024.45629)
Supplement: Supplement 1. — eTable 1. Comparison Between Participants Who Were Seen at 9 Years and Those Who Were Not eTable 2. Complete Case Analyses of Neurodevelopmental Outcome Scores at 9 Years Among Children Born MLP Compared With Term-Born Children eTable 3. Complete Case Analyses of Impairment Rates at 9 Years Among Children Born MLP Compared With Term-Born Children eTable 4. Univariable Associations Between Early-Life Factors and Outcomes at 9 Years for Children Born MLP eFigure 1. Directed Acyclic Graph for 9-Year Outcomes Depicting Assumed Causal Relationships Between Variables eFigure 2. Missingness-Directed Acyclic Graph Depicting the Assumptions Made About the Causes of Missing Data eFigure 3. Cognitive and Academic Scores by Birth Group [file jamanetwopen-e2445629-s001.pdf]

Supplementary Online Content

Cheong JLY, Mainzer RM, Doyle LW, et al. Neurodevelopment at age 9 years among children born at 32 to 36 weeks' gestation. *JAMA Netw Open*. 2024;7(11):e2445629. doi:10.1001/jamanetworkopen.2024.45629

eTable 1. Comparison Between Participants Who Were Seen at 9 Years and Those Who Were Not
eTable 2. Complete Case Analyses of Neurodevelopmental Outcome Scores at 9 Years Among Children Born MLP Compared With Term-Born Children
eTable 3. Complete Case Analyses of Impairment Rates at 9 Years Among Children Born MLP Compared With Term-Born Children
eTable 4. Univariable Associations Between Early-Life Factors and Outcomes at 9 Years for Children Born MLP
eFigure 1. Directed Acyclic Graph for 9-Year Outcomes Depicting Assumed Causal Relationships Between Variables
eFigure 2. Missingness-Directed Acyclic Graph Depicting the Assumptions Made About the Causes of Missing Data
eFigure 3. Cognitive and Academic Scores by Birth Group

This supplementary material has been provided by the authors to give readers additional information about their work.

© 2024 Cheong JLY et al. *JAMA Network Open*.

eTable 1 Comparison between participants who were seen at 9 years and those who were not

Variable	MLP group		Term group	
	Seen n=159	Not seen n=42	Seen n=137	Not seen n=64
Maternal age (year), mean (SD)	34·3 (4·7)	32·8 (5·0)	33·7 (4·6)	32·4 (4·7)
Maternal preeclampsia	29 (18·2)	7 (16·7)	2 (1·5)	1 (1·6)
Assisted conception	31/156 (19·9)	8 (19·1)	16 (11·7)	4 (6·3)
Gestational age at birth (weeks), mean (SD)	34·3 (1·2)	34·7 (1·2)	39·9 (1·2)	39·6 (1·2)
Antenatal corticosteroid exposure	94 (59·1)	27 (64·3)	4 (2·9)	2 (3·1)
Antenatal magnesium sulphate	12 (7·6)	3 (7·1)	0/136 (0)	0 (0)
Multiple birth	55 (34·6)	19 (45·2)	2 (1·5)	0 (0)
Cesarean birth	110 (69·2)	27 (64·3)	51 (37·2)	26 (40·6)
Male sex	72 (45·3)	26 (61·9)	75 (54·7)	32 (50·0)
Birth weight (g), mean (SD)	2156 (436)	2180 (558)	3567 (455)	3476 (428)
Birth weight z-score, mean (SD)	-0·29 (0·97)	-0·48 (1·21)	0·25 (0·84)	0·17 (0·87)
Apgar score @ 5 min, median (IQR)	9 (8, 9)	9 (8, 9)	9 (9, 9)	9 (9, 10)
Any respiratory support	22 (13·8)	6 (14·3)	0 (0)	0 (0)
Neonatal hospitalization (d), median (IQR)	21 (14, 28) n=151	20 (13, 26)	3 (2, 3) n=135	3 (2, 3)
Higher social risk at 2 years	50/157 (31·9)	16/38 (42·1)	29/131 (22·1)	19/53 (35·9)

All data are n (%), unless otherwise specified.
MLP - moderate or late preterm; SD – standard deviation; IQR – interquartile range.

© 2024 Cheong JLY et al. *JAMA Network Open*.

eTable 2 Complete case analyses of neurodevelopmental outcome scores of children born MLP at 9 years compared with term-born children

**Outcome**	**Adjusted mean or median difference (95% CI), p value**
**General intelligence:**	
Full scale IQ	-4·3 (-7·6, -0·9), p=0·01
Verbal comprehension index	-4·0 (-7·3, -0·6), p=0·02
Visual spatial index	-6·2 (-10·2, -2·2), p=0·002
Fluid reasoning index	-2·8 (-6·3, 0·7), p=0·12
Working memory index	-4·4 (-7·7, -1·1), p=0·01
Processing speed index	-0·6 (-4·7, 3·5), p=0·79
**Academic achievement:**	
Reading (single word)	-3·1 (-6·6, 0·5), p=0·09
Reading (pseudoword reading)	-4·1 (-6·9, -1·3), p=0·004
Spelling	-2·2 (-6·2, 1·7), p=0·27
Mathematics	-5·2 (-9·3, -1·1), p=0·01
**Motor function:**	
Movement ABC-2 standard score	-0·5 (-1·3, 0·4), p=0·25
Balance	0.1 (-0.9, 1.0), p=0.91
Aiming and catching	-0.2 (-0.9, 0.6), p=0.63
Manual dexterity	-0.8 (-1.7, 0.1), p=0.08
Behavioral problems:	
Emotional symptoms	0 (-0·9, 0·9), p=1·00
Conduct problems	0 (-0·5, 0·5), p=1·00
Hyperactivity/inattention	0·8 (0·1, 1·5), p=0·02
Peer relationship problems	0 (-0·7, 0·7), p=1·00
Prosocial behavior	0 (-1·0, 1·0), p=1·00
Total difficulties score	1·3 (-0·2, 2·8), p=0·08
**Communication:**	
Total SCQ score	0 (-0·9, 0·9), p=1·00

MLP - moderate or late preterm, SD – standard deviation, IQR – interquartile range, CI – confidence interval, SCQ - Social Communication Questionnaire.
Risk ratios (RR) and risk differences (RDs), adjusted for social risk and multiple pregnancy as informed by the Directed Acyclic Graph (Figure 1).

© 2024 Cheong JLY et al. *JAMA Network Open*.

eTable 3 Complete case analyses of impairment rates of children born MLP at 9 years compared with term-born children

Outcome	Adjusted RR (95% CI), p value	Adjusted RD % (95% CI), p value
No impairment[a]	0·83 (0·61, 1·12)<br>p=0·23	-7·8 (-20·3, 4·7)<br>p=0·22
General intelligence:		
Full scale IQ <-1 SD[b]	1·21 (0·71, 2·08)<br>p=0·48	-3·2 (-6·5, 13·0)<br>p=0·52
Academic performance:		
Reading (single word) <-1 SD[b]	1·31 (0·77, 2·22)<br>p=0·32	-5·0 (-4·9, 15·0)<br>p=0·32
Reading (pseudoword decoding) <-1 SD[b]	1·74 (0·89, 3·40)<br>p=0·11	-7·4 (-0·9, 15·7)<br>p=0·08
Spelling <-1 SD[b]	1·33 (0·81, 2·20)<br>p=0·26	-5·3 (-5·1, 15·7)<br>p=0·32
Mathematics <-1 SD[b]	1·62 (0·91, 2·87)<br>p=0·10	8·7 (-0·3, 19·6)<br>p=0·12
Any academic impairment[c]	1·18 (0·87, 1·60)<br>p=0·29	7·1 (-6·0, 20·3)<br>p=0·29
Motor outcomes:		
Motor impairment[d]	0·96 (0·52, 1·78)<br>p=0·90	-0·8 (-10·3, 8·7)<br>p=0·87
Behavioral problems:		
Any difficulties	1·50 (0·98, 2·28)<br>p=0·06	11·3 (0·2, 22·4)<br>p=0·04
Communication:		
At risk of autism[e]	1·28 (0·49, 3·40)<br>p=0·61	1·7 (-4·1, 7·5)<br>p=0·56

MLP - moderate or late preterm, SD – standard deviation, RR - risk ratio, RD – risk difference, CI – confidence interval, DCD – developmental coordination disorder.
Risk ratios or risk differences adjusted for social risk and multiple pregnancy as informed by the Directed Acyclic Graph (Figure 1).
[a]No impairment in either cognitive, academic performance, motor, behavior or social communication as defined in the text; [b]Relative to term-born controls; [c]Impairment in either one of the four academic domains (single word reading, pseudoword decoding, spelling, mathematics); [d]Any of cerebral palsy or Movement ABC-2 ≤ 5th centile (note there were no children with cerebral palsy in both groups); [e]Social Communication Questionnaire score ≥ 15.

© 2024 Cheong JLY et al. *JAMA Network Open*.

eTable 4 Univariable associations between early life factors and outcomes at 9 years for MLP children

	Cognitive impairment	Academic impairment	Motor impairment	Behavior difficulties	Social communication
	OR (95% CI)	OR (95% CI)	OR (95% CI)	OR (95% CI)	MD (95% CI)
Assisted conception	1.24 (0.46, 3.36)	0.70 (0.31, 1.54)	0.75 (0.20, 2.89)	0.19 (0.05, 0.67)	-1.75 (-3.62, 0.13)
Antenatal corticosteroids	1.81 (0.76, 4.31)	1.64 (0.82, 3.30)	3.29 (1.02, 10.7)	2.88 (1.35, 6.17)	1.31 (-0.28, 2.91)
Gestational age$	1.20 (0.81, 1.77)	1.03 (0.78, 1.36)	0.86 (0.50, 1.48)	0.88 (0.65, 1.17)	-0.82 (-1.58, -0.06)
Multiple birth	0.89 (0.36, 2.22)	0.80 (0.37, 1.72)	0.79 (0.26, 2.41)	0.88 (0.40, 1.93)	-1.68 (-3.26, -0.09)
Male	1.08 (0.47, 2.50)	0.58 (0.30, 1.14)	1.73 (0.67, 4.48)	1.71 (0.89, 3.29)	0.78 (-0.70, 2.26)
Birthweight z-score@	0.87 (0.59, 1.28)	1.01 (0.74, 1.39)	0.67 (0.43, 1.05)	0.74 (0.53, 1.03)	-0.49 (-1.45, 0.46)
Respiratory support	1.41 (0.52, 3.83)	1.33 (0.56, 3.16)	1.71 (0.43, 6.80)	1.12 (0.42, 2.97)	-0.38 (-2.26, 1.50)
Higher social risk	3.28 (1.38, 7.79)	1.78 (0.84, 3.74)	1.95 (0.68, 5.64)	1.46 (0.68, 3.15)	2.19 (-0.05, 4.43)
Developmental delay at 2 years	3.25 (1.35, 7.84)	1.99 (1.01, 3.95)	4.01 (1.47, 10.9)	2.80 (1.40, 5.59)	2.28 (0.55, 4.00)

MLP – moderate or late preterm, OR – odds ratio, CI – confidence interval, MD – mean difference

$OR or MD per week increase in gestational age

@OR or MD per z-score increase in birthweight z-score

© 2024 Cheong JLY et al. *JAMA Network Open*.

eFigure 1 Directed acyclic graph for nine-year outcomes depicting assumed causal relationships between variables

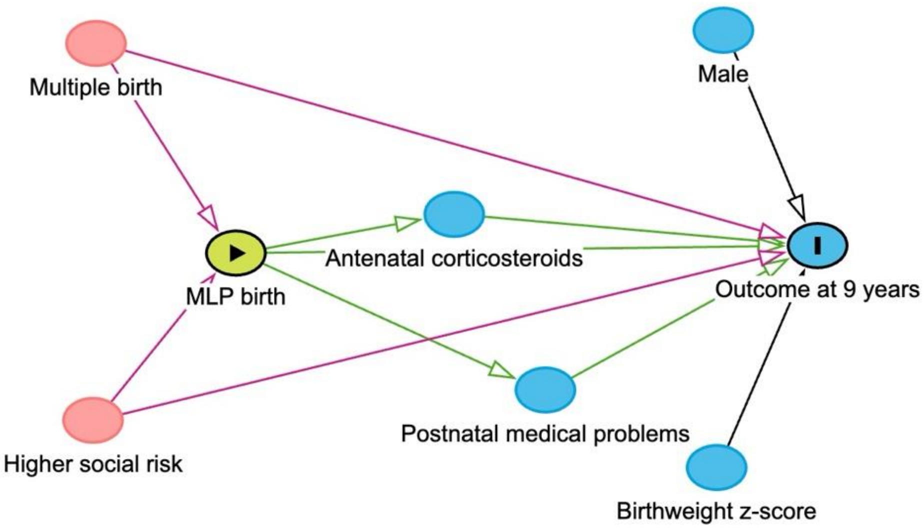

Postnatal medical problems include received respiratory support after birth.

© 2024 Cheong JLY et al. *JAMA Network Open*.

eFigure 2 Missingness-directed acyclic graph depicting the assumptions made about the causes of missing data

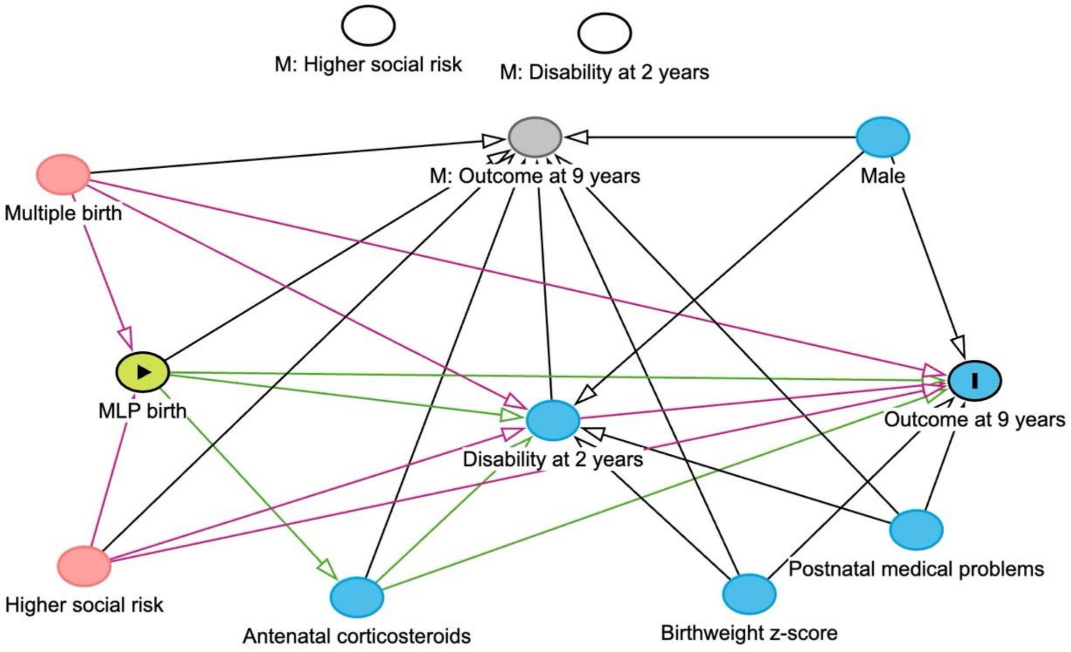

Under the assumptions depicted in this missingness-DAG, the effect of MLP birth on outcomes at 9 years can be consistently estimated from the patterns and associations in the observed data. While a complete case analysis will be biased, a multiple imputation procedure is expected to reduce bias. “M” nodes indicate missingness indicators for incomplete variables.

Variables included in the multiple imputation procedure are: MLP birth, multiple birth, higher social risk, perinatal, and neonatal variables (male sex, birthweight z-score, antenatal corticosteroids, postnatal medical problems [received respiratory support after birth]), disability status at 2 years, and the relevant outcome at 9 years. % missingness was 5.2% for disability at 2 years, and 3.7% for higher social risk. All other imputed variables were complete.

Continuous variables were imputed using linear regression. Binary variables were imputed using logistic regression.

© 2024 Cheong JLY et al. *JAMA Network Open*.

eFigure 3 Cognitive and academic scores by birth group

Mean and 95% confidence intervals are superimposed in black.
Reference mean refers to published normative means of 100.
MLP – moderate of late preterm

eFigure 3a Full scale IQ

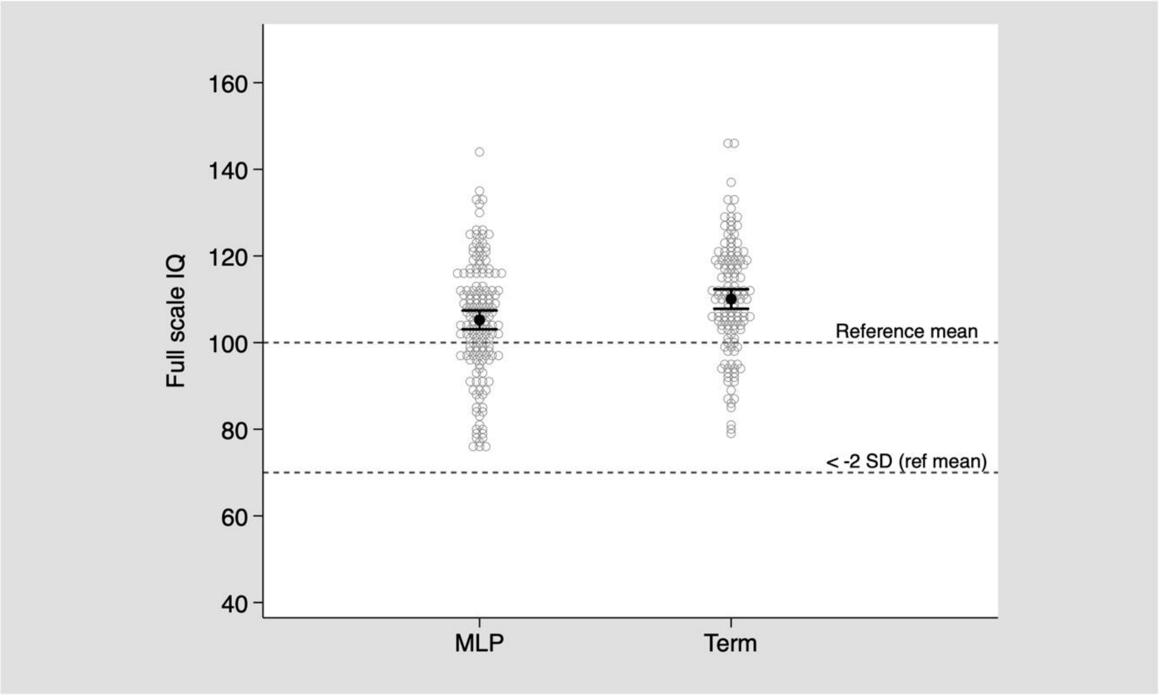

eFigure 3b Reading (single word)

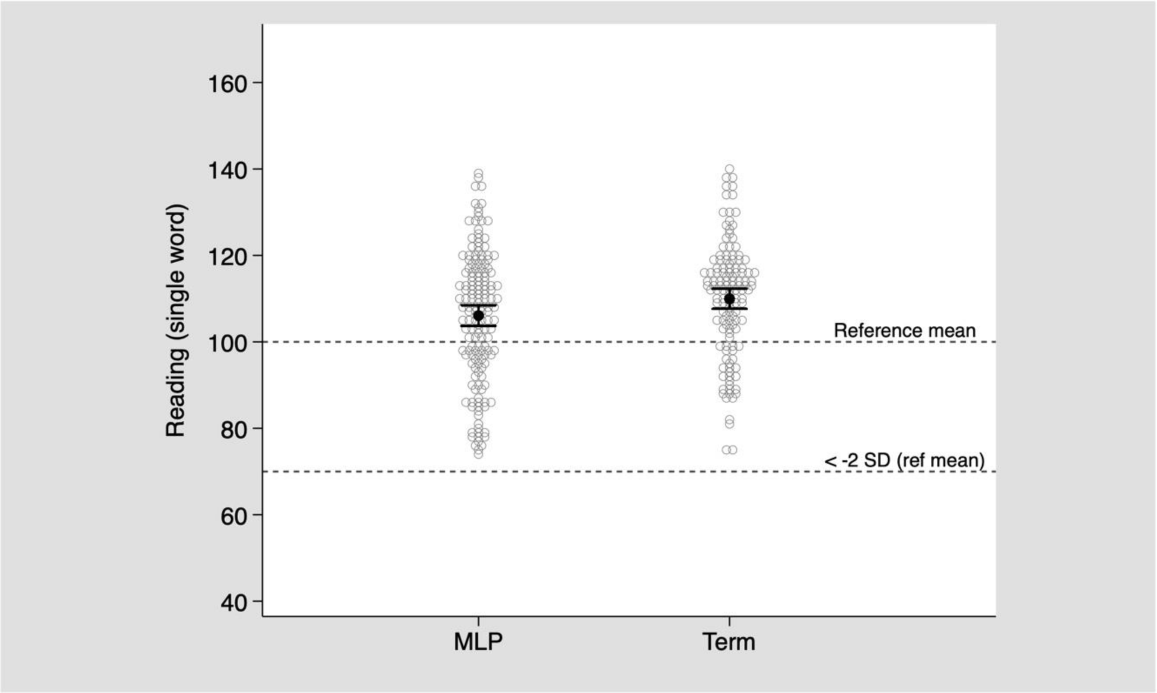

© 2024 Cheong JLY et al. *JAMA Network Open*.

eFigure 3c Reading (Pseudoword decoding)

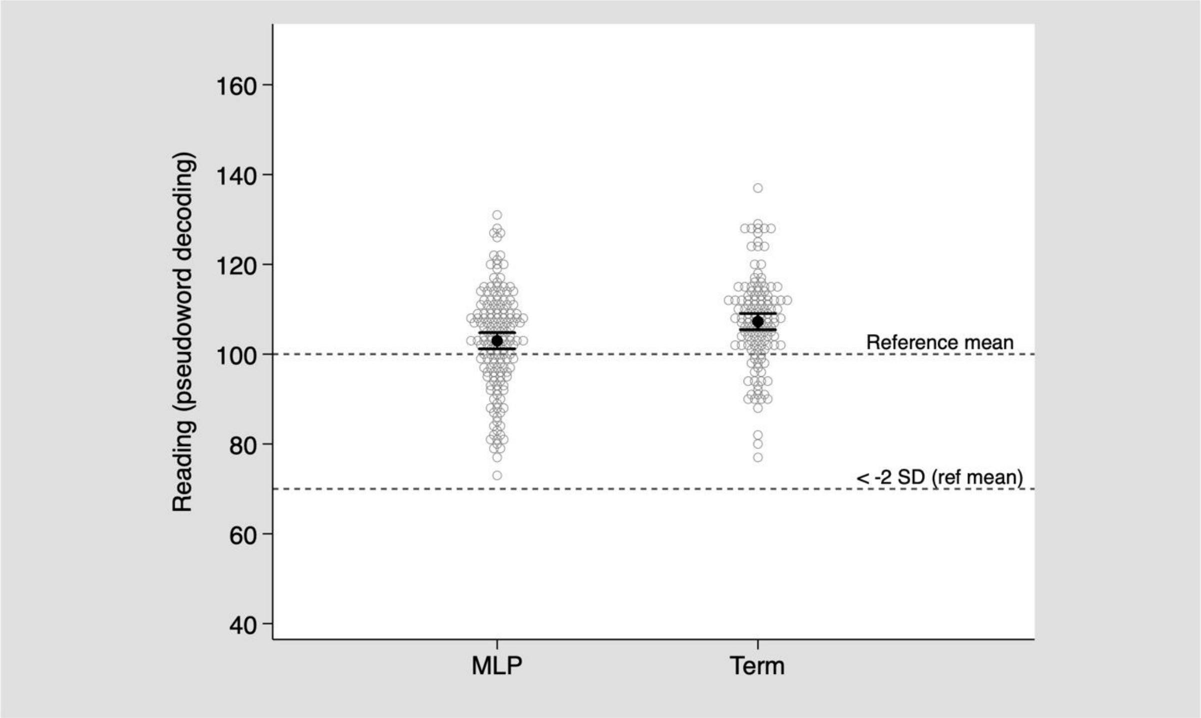

eFigure 3d Spelling

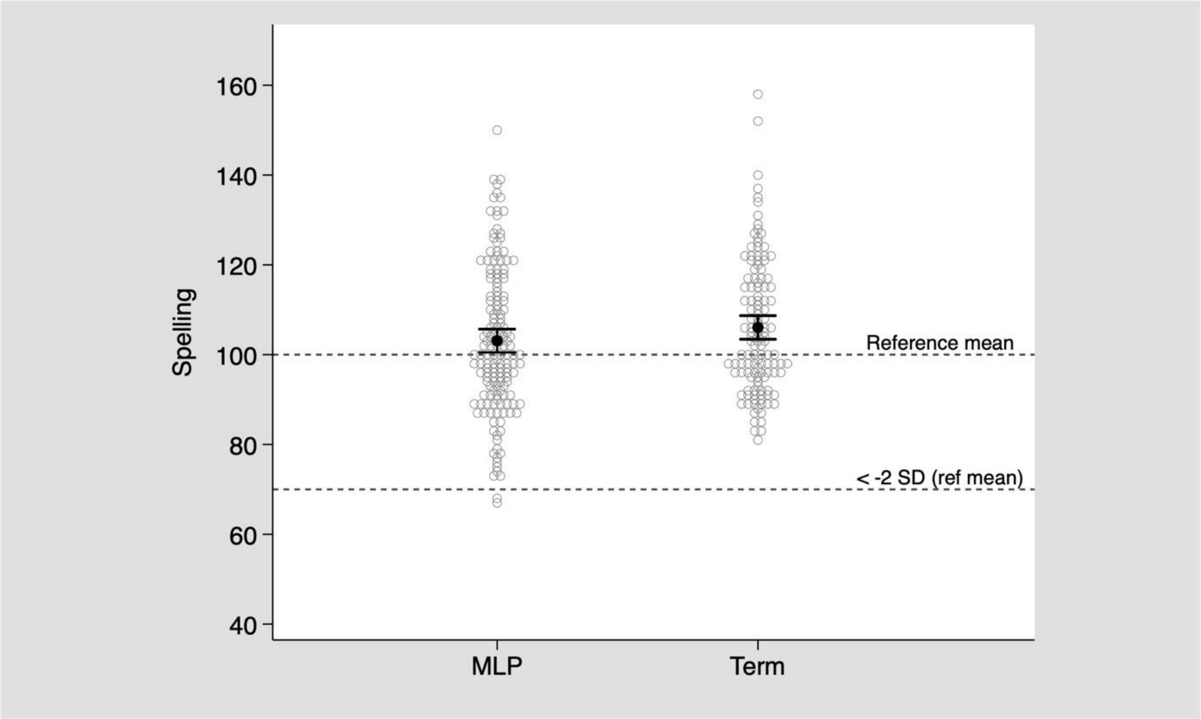

© 2024 Cheong JLY et al. *JAMA Network Open*.

eFigure 3e Mathematics

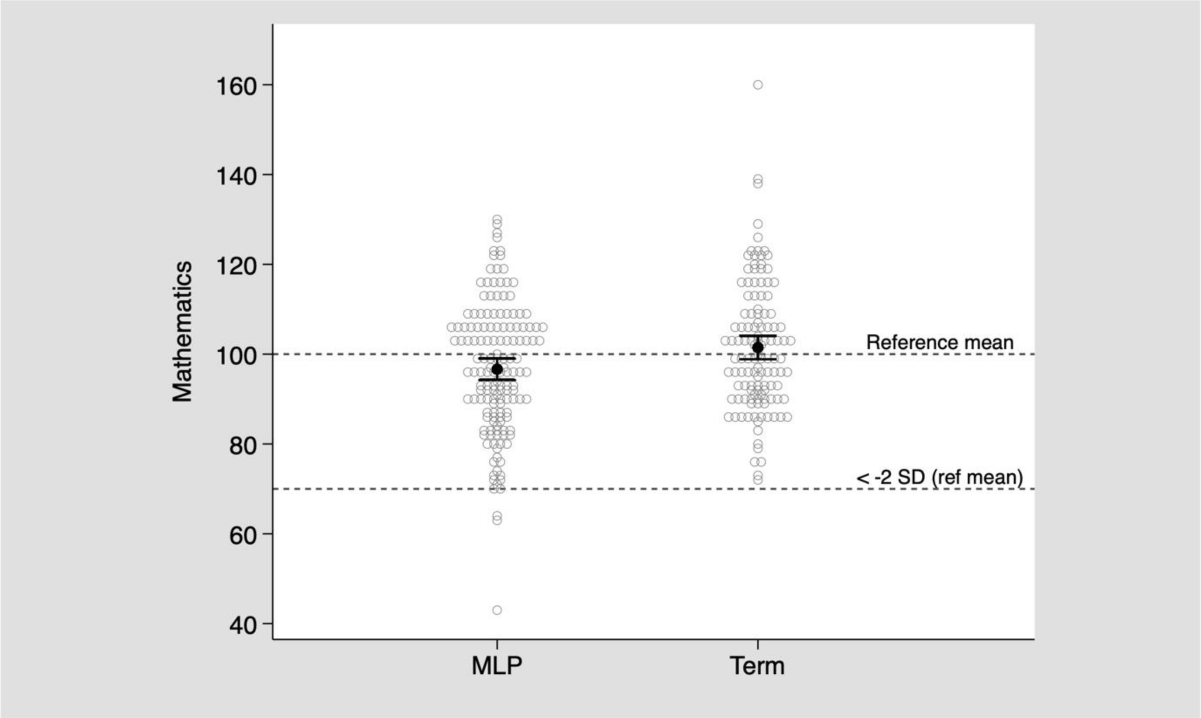

© 2024 Cheong JLY et al. *JAMA Network Open*.
