# Supplementary material for: Neurodevelopment at Age 9 Years Among Children Born at 32 to 36 Weeks’ Gestation
Source: JAMA Netw Open. 2024 Nov 18;7(11):e2445629. doi: 10.1001/jamanetworkopen.2024.45629 (PMC11574691; doi:10.1001/jamanetworkopen.2024.45629)
Supplement: Supplement 2. — Data Sharing Statement [file jamanetwopen-e2445629-s002.pdf]

Data Sharing Statement

Cheong. Neurodevelopment at Age 9 Years Among Children Born at 32 to 36 Weeks' Gestation. *JAMA Netw Open*. Published November 18, 2024.
doi:10.1001/jamanetworkopen.2024.45629

Data
Data available: Yes
Data types: Deidentified participant data
How to access data: The data will be available on request to the corresponding author, with consideration given to the protocol of the proposed project.
When available: With publication

Supporting Documents
Document types: None

Additional Information
Who can access the data: NA
Types of analyses: For a specified purpose depending on the individual request.
Mechanisms of data availability: After approval of a proposal and with a signed data access agreement.
